# Role of Silicon in Mediating Phosphorus Imbalance in Plants

**DOI:** 10.3390/plants10010051

**Published:** 2020-12-29

**Authors:** An Yong Hu, Shu Nan Xu, Dong Ni Qin, Wen Li, Xue Qiang Zhao

**Affiliations:** 1 School of Geographical Science, Nantong University, Nantong 226019, China; ayhu2018@ntu.edu.cn (A.Y.H.); 1822011014@stmail.ntu.edu.cn (S.N.X.); 1822061004@stmail.ntu.edu.cn (D.N.Q.); 1822011015@stmail.ntu.edu.cn (W.L.); 2 State Key Laboratory of Soil and Sustainable Agriculture, Institute of Soil Science, Chinese Academy of Sciences, Nanjing 210008, China; 3 University of Chinese Academy of Sciences, Beijing 100049, China

**Keywords:** silicon, silicon transporter, phosphorus transporter, phosphorus imbalance, phosphorus deficiency, excess phosphorus

## Abstract

The soil bioavailability of phosphorus (P) is often low because of its poor solubility, strong sorption and slow diffusion in most soils; however, stress due to excess soil P can occur in greenhouse production systems subjected to high levels of P fertilizer. Silicon (Si) is a beneficial element that can alleviate multiple biotic and abiotic stresses. Although numerous studies have investigated the effects of Si on P nutrition, a comprehensive review has not been published. Accordingly, here we review: (1) the Si uptake, transport and accumulation in various plant species; (2) the roles of phosphate transporters in P acquisition, mobilization, re-utilization and homeostasis; (3) the beneficial role of Si in improving P nutrition under P deficiency; and (4) the regulatory function of Si in decreasing P uptake under excess P. The results of the reviewed studies suggest the important role of Si in mediating P imbalance in plants. We also present a schematic model to explain underlying mechanisms responsible for the beneficial impact of Si on plant adaption to P-imbalance stress. Finally, we highlight the importance of future investigations aimed at revealing the role of Si in regulating P imbalance in plants, both at deeper molecular and broader field levels.

## 1. Introduction

Silicon (Si) is the second most abundant element in the Earth’s crust, and its content within plants ranges from 0.1% to 10% depending on species. Although still not proven to be a plant-essential element, Si is widely recognized as a beneficial factor for plant growth and development. Si can alleviate biotic stresses, such as plant pathogens and insect pests, and abiotic stresses, such as drought, heat, cold, lodging, salinity, ultraviolet radiation, metal toxicity and nutrient imbalance [1]. Si can strengthen plant resistance to abiotic and biotic stresses via physical and physiological biochemical mechanisms. Recently, several authors have systematically reviewed research progress on the elucidation of mechanisms of Si-mediated alleviation of biotic and abiotic stresses in plants [2,3,4,5,6,7]. In these reviews, however, the beneficial effects of Si on nutrient imbalance have received relatively little attention. Because of increasing deficiencies or excesses of some essential elements in soils worldwide, the importance of Si in mitigating nutrient imbalance has gradually attracted much attention.

Phosphorus (P) is a key element that greatly influences plant growth and productivity. Available P is very low in soils for two reasons: P fixation into organic forms, and binding or adsorption of P either by aluminum and iron (Fe) oxides or calcium minerals, depending on soil pH [8]. Critical strategies for improving crop performance in low-P soils are the improvement of P acquisition efficiency by enhancing soil P availability and the enhancement of P utilization efficiency in inner plant organs. Excess-P stress is also observed in some greenhouse soils subjected to heavy application of P fertilizer or in hydroponic culture where a high P concentration is supplied [9]. The beneficial effect of Si on P deficiency or excess stress has been reported for a number of plant species [10,11,12,13,14,15,16,17,18,19,20,21,22,23,24,25]. To the best of our knowledge, however, no review has discussed the roles and mechanisms of Si in alleviating P-imbalance stress in plants. In this review, therefore, we focus on the roles of Si in mitigating P-deficiency or excess-P stress. We also discuss the mechanisms underlying Si enhancement of plant tolerance to P-imbalance stress.

## 2. Silicon (Si) Absorption, Transport and Accumulation in Higher Plants

All plants contain Si [26]. In some plants, termed Si-accumulator plants, concentrations of Si may be almost as high as those of macro-elements. The leaf is the organ with the highest Si accumulation, with Si generally accounting for more than 1.5% of leaf dry weight in Si-accumulator plants. Si is taken up and translocated by plants in the form of uncharged silicic acid (H_4_SiO_4_), which is ultimately irreversibly precipitated as amorphous silica in cell walls and extracellular spaces by transpirational flow. The Si content of various plant species differs depending on their Si absorption capabilities and Si loading capacities from roots to xylem [27].

Although the cytoplasmic membrane of plant roots may provide a channel for the absorption of silicic acid by non-ionic diffusion, the permeability coefficient of silicic acid through the plasma membrane is only 10^−10^ m·s^−1^ [28]. This proposed route is, therefore, not consistent with the characteristics of some Si-accumulating plants, especially graminaceous ones [29]. Absorption of Si by plants and its transport to the xylem involve both passive and active uptake processes [30]. Si uptake by plants is significantly inhibited by treatment with metabolic inhibitors (2,4-dinitrophenol and potassium cyanide) or low temperature stress, thus demonstrating that plants can actively absorb and transport Si [29,30,31]. Plant roots are able to take up Si from an external medium and transfer it to cortical cells via a low-affinity silicic acid transporter with a Km value of 0.15 mM. In one kinetic study, differences in Si uptake by rice (*Oryza sativa*), cucumber (*Cucumis sativus*) and tomato (*Solanum lycopersicum*) were found to be correlated with the abundance of Si transporters on the root plasma membrane, which was highest and lowest in rice and tomato, respectively [30].

The identification of two Si transporter genes (*OsLsi1* and *OsLsi2*) in rice was a landmark discovery that accelerated the development of the field of plant Si research [32,33]. OsLsi1 belongs to the nodulin 26-like intrinsic protein (NIP) subfamily of aquaporins, which is responsible for Si uptake from soil into root cells. In rice, OsLsi1 is an influx Si transporter located on the distal side of both exodermis and endodermis in roots. OsLsi2 is a putative anion and efflux transporter located on the proximal side of endodermis and exodermis in the root plasma membrane. According to a mathematical model based on a simple diffusion equation, this characteristic cellular localization pattern explains the high Si uptake capacity of rice [34]. A number of genes involved in Si uptake and distribution (*Lsi1*, *Lsi2*, *Lsi3* and *Lsi6*) have now been identified and functionally validated in numerous plant species, both monocots, such as rice, maize (*Zea mays*) and barley (*Hordeum vulgare*), and dicots, such as pumpkin (*Cucurbita moschata*), cucumber, tomato and horsetail (*Equisetum arvense*) (Table 1) [32,33,35,36,37,38,39,40,41,42,43,44,45,46,47]. Depending on the plant species, these genes are expressed in roots, shoots or both organs (Table 1). The tissue and cellular locations of these genes, which differ among plant species, determine the different roles of Si transporters in plant Si uptake, transport and accumulation.

## 3. Phosphorus (P) Uptake and Utilization by Plants

One of the most essential mineral elements, P is not only a component of many important compounds in plants, such as nucleic acids, proteins and phospholipids, but also plays an important role in photosynthesis, respiration and many enzymatic reactions [48]. P homeostasis in plants is, therefore, indispensable for normal physiological and biochemical functioning, but most P in soils is biologically unavailable because of its poor solubility, strong sorption and slow diffusion [49]. Approximately 30% of the world’s agricultural soils are estimated to be P deficient [50]. To ensure crop productivity and quality, farmers often increase the amount of P fertilizer applied to P-deficient soils. Unfortunately, the P use efficiency of applied P fertilizer is less than 20%, with the residual P easily immobilized in soil or flowing into surface water through runoff [51]. In addition, rock P, a non-renewable resource and the main source of P fertilizer, is estimated to become exhausted within the next 50–100 years [52].

P often exists in soil in two chemical forms: organic and inorganic (orthophosphate, Pi). Pi acquisition, utilization and homeostasis depend on complicated transport processes mediated by Pi transporters belonging to five phosphate transporter (PHT) families: PHT1 to PHT5 [53,54,55,56]. Plant PHT1 family proteins, whose role in P uptake, mobilization and re-utilization has been widely studied, belong to a major facilitator superfamily (MFS). P transport characteristics and kinetic parameters (Km) differ significantly among PHT1 family members. Many PHT1 family members have been identified in higher plants, including Arabidopsis (*Arabidopsis thaliana*), rice, wheat (*Triticum aestivum*), maize, barley, soybean (*Glycine max*), sorghum (*Sorghum bicolor*), tomato and potato (*Solanum tuberosum*) [57]. Most PHT1 family genes are induced by Pi starvation and expressed mainly in plant roots, with the expression of a few family members also detectable in other plant organs [53,58,59,60]. Some MYB and WRKY transcriptional factors and *ZAT6* can bind to various *cis*-elements, such as MYCS, P1BS and W-box elements, in the promoter region of plant PHT1 family genes [61,62,63,64,65] to regulate *PHT1* gene expression. PHT1 family genes are also affected by arbuscular mycorrhiza [55,66,67]. This post-transcriptional gene regulation is also crucial for proper plant responsiveness to Pi. PHF1 proteins, SPX domain-containing proteins, microRNAs, and phosphorylation/dephosphorylation proteins are also involved in the post-transcriptional regulation and signal transduction of PHT1 family genes [68,69,70,71,72,73,74], as are plant hormones such as auxin, ethylene and cytokinin [75,76,77,78].

PHT1 proteins, which generally function in the acquisition of Pi from soil, have received far more attention than other PHT family genes. PHT2, PHT3, PHT4 and PHT5 family members, which play vital roles in maintaining Pi distribution and homeostasis in plants, are localized in plastids [79,80], mitochondria [54], the plastid envelope or the Golgi apparatus [81] and vacuolar membranes [82], respectively. The chloroplast envelope-localized protein PHT2 is responsible for Pi translocation into leaves and Pi starvation responses [79,83]. PHT3 family members, which are plant mitochondrial Pi transporters, are involved in Pi exchange between the cytoplasm and the mitochondrial matrix via the Pi/H^+^ symport or Pi/OH^−^ antiport [84,85]. The plastid and Golgi apparatus-located *PHT4* genes play important roles in various biological processes, including Pi translocation in plastids and the Golgi apparatus [81,86], carbon metabolism [87,88], pathogen resistance [89], and salt tolerance [90]. SYG1/PHO81/XPR1-MFS (SPX-MFS) proteins, which constitute the PHT5 family, function in Pi sequestration in vacuoles and the transport of Pi across the tonoplast in plants [82,91].

## 4. Mechanisms of Si Alleviation of P-Deficiency Stress in Plants

The first evidence of Si-alleviated P deficiency was obtained from a long-term field experiment conducted at the Rothamsted Experimental Station. In that experiment, which compared barley yields in two fields not subjected to P fertilizer application, the yield of barley fertilized with Si was higher than that of a field without Si amendment [18]. The beneficial effect of Si on P-deficiency stress has been subsequently reported in several graminaceous species, such as wheat, rice and maize, under soil cultivation as well as hydroponic conditions (Table 2). It is known that large amounts of P exist in soils bound in unavailable form [92,93]. The effect of Si on P deficiency was initially thought to be associated with the enhancement of P availability in soil. A significantly positive correlation existed between Si availabilities and P mobilization in 143 representative Artic soils [94]. Furthermore, exogenous Si also was proved to contribute to the increased P activization from Fe-P phases on mineral surfaces [94,95]. Many studies have shown that the increased soil P availability under high Si can be explained by Si competition with P for binding at the surface of soil minerals resulting in P mobilization [96,97,98,99,100,101,102]. Several studies suggest that the competitive ability of Si against P may be pH-dependent [21,103,104,105]. The effect of Si on soil P mobilization may depend on Si fertilizer form, Si level, soil mineralogy and plant P uptake.

Si is commonly applied as calcium or sodium silicate, both of which increase soil pH. In acidic soils, aluminum (Al) toxicity is the most important factor limiting crop growth and production. At soil pH values at or below pH 5, Al ions are solubilized into the soil solution and dramatically inhibit root growth and function, in turn severely impairing water and nutrient acquisition by roots and, thereby, leading to a significant reduction in crop yields [8,106,107]. An increase in soil pH due to Si application can effectively improve the growth condition of plant roots suffering from Al toxicity [24], thus resulting in increased transpiration and, therefore, greater P uptake and utilization [21]. Furthermore, high soil pH, within a certain range, can facilitate soil P activization and plant P uptake [108].

Plants have evolved diverse strategies to cope with P deficiency. One effective strategy to counteract P-deficiency stress is the secretion of organic acid anions (e.g., malate, citrate and oxalate) by plant roots [109]. Current evidence indicates that Si application strongly promotes the exudation of both malate and citrate by roots, with the amounts of these anions released by roots always higher than in other tested treatments [24]. The highest efflux of malate and citrate in response to P deficiency occurs following Si treatment. Furthermore, Si has been reported to upregulate the expressions of Pi transporter genes (*TaPHT1;1* and *TaPHT1;2*) in wheat roots at low P [24]. Despite these findings, the mechanisms underlying Si modulation of Pi transporter gene expression in roots require further study. At low P levels, in fact, the greatest beneficial effect of Si on plant growth, improvement of internal P use efficiency, occurs indirectly via a decrease in Fe and manganese (Mn) uptake [14]. Si-decreased Mn accumulation in shoots is due to the decrease of Mn translocation from roots to shoots by the formation of Mn-Si complex in root cells, and down-regulating the expression of Mn transporter gene [110]. Given that P usually has a high affinity to metals such as Fe and Mn [9], the internal availability of P in plants may be affected by levels of Mn, Fe and other metals when the P concentration is low.

## 5. Mechanisms of Si-Based Alleviation of Excess-P Stress in Plants

Excess-P stress rarely occurs in natural soils. In greenhouse production systems, however, famers apply high levels of P fertilizer, which results in large amounts of P accumulated in the soil [111]. Soil Olsen-P, an important indicator of soil P supply ability and the risk of soil P runoff and leaching into the environment, has reached as high as 200 mg P kg^−1^ in greenhouse systems (compared with the critical value of 46.0–58.0 mg P kg^−1^ in vegetable field soils) [112,113,114]. Although accumulated P is required for metabolism and storage in plants, high P concentrations inhibit enzymatic reactions, create abnormal osmotic pressure, and decrease essential metal element availability in cells [115]. For example, excess P application has been shown to reduce zinc (Zn) uptake and bioavailability [116,117,118,119]. Recent studies suggest that the Zn concentration in aerial parts of plants is negatively related to soil-available P in both calcareous and acid soils, while a high P concentration in soil has a strong antagonistic effect on Zn accumulation in plants [120,121]. In plants, typical symptoms of P-induced Zn deficiency are leaf chlorosis and necrosis [122]. Furthermore, low Zn levels in plants that are important nutritional sources of this element, such as cereal grains and vegetables, may lead to inadequate Zn intake in humans.

In several plant species, Si alleviation of excess P damage has been attributed to a decrease in P uptake (Table 2) that reduced the concentration and accumulation of inorganic P in plants. In one hydroponic experiment, Si significantly decreased P uptake by rice under high P levels, with the amount of inorganic P in shoots almost half that of shoots cultivated without supplemental Si [14]. Si has been found to decrease P uptake in rice as well as some Si non-accumulating plants, such as tomato, soybean and strawberry (*Fragaria vesca*) [27]. Furthermore, application of Si to cucumber, a moderate Si accumulator, has also been found to alleviate symptoms of toxicity induced by P excess, resulting in a marked decrease in leaf P and an increase in the proportion of water-extractable Zn [15].

One possible explanation for the aforementioned observations is that Si deposition in endodermal cells of plant roots may contribute to decreased P uptake and alleviation of excess-P stress. This effect has been attributed to the formation of apoplastic barriers to P permeability across roots caused by Si deposition in roots, which decreases excessive uptake of P [9,123]. Another explanation is that the formation of a cuticle-silica double layer due to Si deposition in leaves reduces the plant transpiration rate. Transpiration is negatively correlated with the Si content of aerial parts of rice, and the rice transpiration rate can be reduced by 20–30% when the SiO_2_ content of shoots exceeds 10% of the dry matter weight [104]. A positive correlation exists between plant leaf P content and transpiration rate [124]. Except for several Si-accumulator plants, however, most plant species accumulate little Si in their organs. In addition to the physical barrier function of Si deposition in roots or leaves, Si may therefore participate in the regulation of plant responses to excess-P stress in other ways.

A number of studies have suggested that Si improves the growth of plants under abiotic stress by regulating the expressions of genes directly associated with the uptake and translocation of stress factors. We recently investigated the effect of Si on P uptake and accumulation under high P concentrations in both relatively long- and short-term experiments. We found that Si decreased both P uptake and accumulation in rice by downregulating the P transporter gene *OsPHT1;6* in roots [16]. A split-root experiment further indicated that *OsPHT1;6* expression was decreased by Si accumulation in shoots, resulting in decreased Pi uptake in rice [16]. In this study, we dissected the physiological and molecular mechanisms underlying the beneficial effects of Si under excess-P stress using the rice mutant *lsi1*, defective in Si uptake. Compared with the wild type, Oochikara, *lsi1* accumulated a similar level of Si in roots but exhibited much less accumulation in shoots [125]. By taking advantage of this mutant, the study has proved the importance of Si accumulation in shoots to alleviate excess-P stress.

In cereal crops, P stored in seeds accounts for 60–85% of total plant P at maturity [126,127,128]. Moreover, approximately 65–85% of total P in grains is stored in the form of phytate [121]. Phytate, which is barely digestible by monogastric animals and humans, combines with some essential microelements, such as Zn and Fe, thus greatly reducing their availability [129]. Additionally, non-digested phytate is excreted into the environment. Decreasing the total P and phytate content of grains is thus an important goal for solving these environmental and nutritional problems [130]. In a field experiment, we observed that wild-type plants accumulated less P in grains, husks and straw compared with the Si uptake-defective mutant *lsi1* [16]. This result can be attributed to a decrease in P uptake in the wild type caused by Si, as higher Si accumulation was observed in shoots of the wild type than in *lsi1* [16]. This finding indicates that Si has an important role in reducing P input to maintain P balance and in increasing the nutritional value of primary products.

## 6. Conclusions and Future Prospects

**Figure 1:**
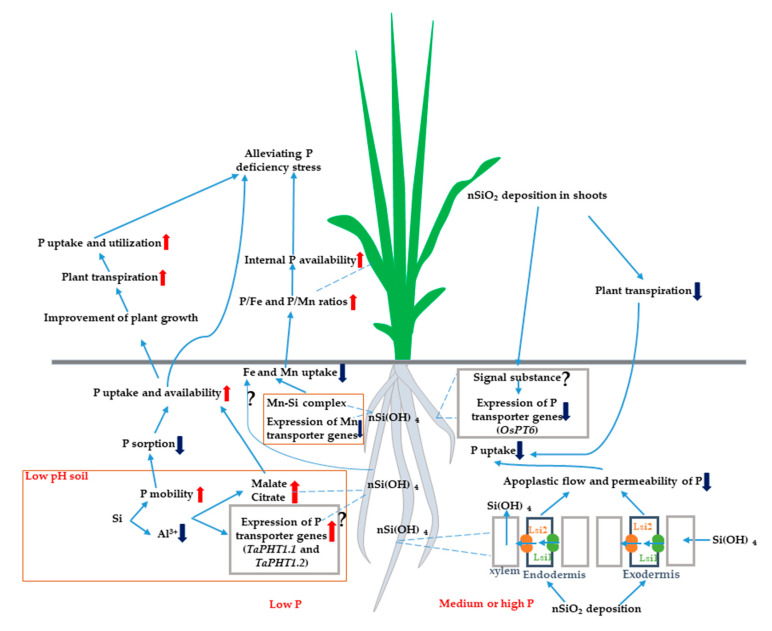
A schematic model for the beneficial impact of silicon on plant under P imbalance stress.

Although not considered an essential element in higher plants, Si has been shown to have beneficial roles in the enhancement of plant resistance to various biotic and abiotic stresses, including diseases, insect pests, drought, salt, heavy metals and nutrient imbalance. Many studies have been conducted to illustrate the mechanisms of Si alleviation of P-deficiency or excess-P stress. These investigations have collectively shown the beneficial effects of Si supplementation on P-imbalance stress in different plant species. In regard to the diversity of the Si-mediated P-imbalance stress resistance mechanisms, the above-described studies in this review have demonstrated that exogenous Si application is able to alleviate P-deficiency stress by increasing P mobility, decreasing exchangeable Al^3+^ in acid soils, increasing the exudation of both malate and citrate, upregulating P transporter genes and enhancing internal P utilization by decreasing Fe and Mn uptake (Figure 1). At the same time, the positive effects of exogenous Si on excess-P stress can be attributed to the formation of apoplastic barriers arising from Si deposition in the cortex cells of roots and also the downregulation of P transporter genes (Figure 1).

Si-mediated tolerance mechanisms against P-deficiency or excess-P stress should continue to contribute to the improvement of P-imbalance stresses in diverse crop plants. Given currently published results, however, research on the improvement of plant P balance via Si has mainly focused on physiology and biochemistry, with deeper molecular regulatory mechanisms remaining to be fully revealed. Therefore, our recommendations regarding future research on Si alleviation of P-imbalance stresses and research prospects are as follows:

(1) With the development of multi-omics methods, such as metabolomics, ionomics, transcriptomics and proteomics, the molecular mechanisms of Si mitigation of plant biotic and abiotic stresses can be explored in depth. These investigations should provide new insights and opportunities for dissecting the underlying mechanisms of Si mediation of P balance in plants under P-deficiency or excess-P stress.
(2) Recent studies have demonstrated experimentally the direct effect of Si on the expressions of P transporter genes (*TaPHT1;1*, *TaPHT1;2* and *OsPHT1;6*), but the associated detailed regulatory mechanisms and signaling pathways have not yet been fully determined.
(3) *lsi1* is a rice mutant defective in Si uptake. Although Si accumulation in the roots of *lsi1* is similar to that of the wild type, the level of Si accumulated in shoots is much lower in the mutant. This characteristic can be applied to help researchers distinguish the different roles of Si accumulation in shoots and roots in Si alleviation of P-imbalance stress.

The adaptive mechanisms of plant response to P-imbalance stress are complex. The regulatory effects of Si on P-deficiency or excess-P stress may constitute a complex system involving various biochemical and physiological processes as well. Consequently, systematic studies of Si-mediated alleviation of P-imbalance stress in plants with a focus on molecular and genetic levels should provide a theoretical foundation for practical applications of Si to agricultural production.

## Figures and Tables

**Table 1:** Genes involved in Si transport in plants.

Plant Species	Gene Name	Function	Spatial Expression	Transcriptional Regulation by Si Supply	References
*Oryza sativa*	*OsLsi1*	Si influx transporter	Distal side of both exodermis and endodermis in roots	Downregulated	[32]
	*OsLsi2*	Si efflux transporter	Proximal side of both exodermis and endodermis in roots; bundle sheath cell layer around enlarged vascular bundles	Downregulated	[33]
	*OsLsi3*	Si efflux transporter	Parenchyma tissues between enlarged vascular bundles and diffuse vascular bundles	Unknown	[35]
	*OsLsi6*	Si influx transporter	Xylem transfer cells located at the outer boundary region of enlarged large vascular bundles in node I	Unknown	[35,36,37]
*Zea mays*	*ZmLsi1*	Si influx transporter	Plasma membrane of the distal side of root epidermal and hypodermal cells in seminal and crown roots; cortex cells in lateral roots	Unaffected	[38]
	*ZmLsi2*	Si efflux transporter	Endodermis with no polarity in roots	Downregulated	[39]
	*ZmLsi6*	Si transporter for xylem unloading	Xylem parenchyma cells closed to vessels in both leaf sheaths and leaf blades	Unaffected	[38]
*Hordeum vulgare*	*HvLsi1*	Si influx transporter	Plasma membrane on the distal side of epidermal and cortical cells; plasma membrane of hypodermal cells in lateral roots	Unaffected	[40]
	*HvLsi2*	Si efflux transporter	Endodermis with no polarity in roots	Downregulated	[39]
	*HvLsi6*	Si uptake in root tips; xylem unloading in leaf blades and sheaths; intervascular transfer in nodes	Epidermis and cortex cells of tips; parenchyma cells of vascular bundles in leaf blades and sheaths; transfer cells surrounding enlarged vascular bundles adjacent to numerous xylem vessels	Unaffected	[41]
*Cucurbita moschata*	*CmLsi1*	Si influx transporter	Plasma membrane of all cells in roots	Unknown	[43]
	*CmLsi2-1; CmLsi2-2*	Si efflux transporter	Roots and shoots	Unknown	[42]
*Cucumis sativus*	*CsLsi1*	Si influx transporter	Distal side of endodermis and cortical cells in root tips as well as root hairs near root tips	Downregulated	[44]
	*CsLsi2*	Si efflux transporter	Endodermal cells of roots without polar distribution	Unknown	[45]
*Solanum lycopersicum*	*SlLsi1*	Si influx transporter	Plasma membrane of both root tips and basal regions without polarity	Unaffected	[46]
*Equisetum arvense*	*EaLsi2-1*	Si efflux transporter	Roots and shoots	Unknown	[47]
	*EaLsi2-2*				

**Table 2:** Summary of effects of silicon (Si) on phosphorus (P) content and plant growth: (−) inhibition, (+) stimulation, and (0) no change.

Taxon	Si Form	P Level	Experimental Conditions	pH of Experimental Medium	Effects on Shoot P Content	Effects on Plant Growth?	References
*Glycine max; Fragaria ananassa; Solanum lycopersicum*	Silicic acid	0.58 mM; 0.23 mM; 2.3 mM;	Hydroponic	5.5	Decreased	+	[10,11,12]
*Oryza sativa*	Silicic acid	1.4–700 µM	Hydroponic	5.5	No effects under low P (≤14 ppm), but decreased at higher P levels	+	[13,14]
*Cucumis sativus*	Silicic acid	0.05–1.2 mM	Hydroponic	5.5	No effects under low P (0.05 mM), but decreased at higher P levels	+	[15]
*Oryza sativa*	Silicic acid	90–210 µM	Hydroponic	5.5	Decreased	+	[16]
*Solanum lycopersicum*	Potassium silicate	0.44–0.66 mM	Hydroponic	5.8–6.0	Increased	+	[17]
*Hordeum vulgare*	Sodium silicate	No P addition	Field	Unknown	Decreased slightly	+	[18]
*Oryza sativa*	Silicic acid	No P addition	Soil culture	4.5	No effects	+	[19]
*Oryza sativa*	Sodium silicate	No P addition	Soil culture	4.5	No effects	+	[20]
*Zea mays*	Calcium silicate	0–0.3 g per 1.5 kg soil	Soil culture	5.3	Increased	+	[21]
*Oryza sativa*	Diatomaceous earth	25–50 kg P ha^−1^	Field	7.4	Increased	+	[22]
*Oryza sativa*	Wollastonite, slag and foliar Si solution	4.5 ton ha^−1^ and 0–80 mg L^−1^	Soil culture	6.1	No effects	0 or −	[23]
*Triticum aestivum*	Sodium silicate	No P addition	Soil culture	4.0	Increased	+	[24]
*Solanum tuberosum*	Powder FertiSilica	10–200 ppm	Soil culture	4.5	No effects	0	[25]
